# Trimming of mammalian transcriptional networks using network component analysis

**DOI:** 10.1186/1471-2105-11-511

**Published:** 2010-10-13

**Authors:** Linh M Tran, Daniel R Hyduke, James C Liao

**Affiliations:** 1 Department of Chemical and Biomolecular Engineering, University of California, Los Angeles, CA 90095-1592, USA

## Abstract

**Background:**

Network Component Analysis (NCA) has been used to deduce the activities of transcription factors (TFs) from gene expression data and the TF-gene binding relationship. However, the TF-gene interaction varies in different environmental conditions and tissues, but such information is rarely available and cannot be predicted simply by motif analysis. Thus, it is beneficial to identify key TF-gene interactions under the experimental condition based on transcriptome data. Such information would be useful in identifying key regulatory pathways and gene markers of TFs in further studies.

**Results:**

We developed an algorithm to trim network connectivity such that the important regulatory interactions between the TFs and the genes were retained and the regulatory signals were deduced. Theoretical studies demonstrated that the regulatory signals were accurately reconstructed even in the case where only three independent transcriptome datasets were available. At least 80% of the main target genes were correctly predicted in the extreme condition of high noise level and small number of datasets. Our algorithm was tested with transcriptome data taken from mice under rapamycin treatment. The initial network topology from the literature contains 70 TFs, 778 genes, and 1423 edges between the TFs and genes. Our method retained 1074 edges (i.e. 75% of the original edge number) and identified 17 TFs as being significantly perturbed under the experimental condition. Twelve of these TFs are involved in MAPK signaling or myeloid leukemia pathways defined in the KEGG database, or are known to physically interact with each other. Additionally, four of these TFs, which are Hif1a, Cebpb, Nfkb1, and Atf1, are known targets of rapamycin. Furthermore, the trimmed network was able to predict *Eno1 *as an important target of Hif1a; this key interaction could not be detected without trimming the regulatory network.

**Conclusions:**

The advantage of our new algorithm, relative to the original NCA, is that our algorithm can identify the important TF-gene interactions. Identifying the important TF-gene interactions is crucial for understanding the roles of pleiotropic global regulators, such as p53. Also, our algorithm has been developed to overcome NCA's inability to analyze large networks where multiple TFs regulate a single gene. Thus, our algorithm extends the applicability of NCA to the realm of mammalian regulatory network analysis.

## Background

### The common decomposition method in the network analysis

Transcriptional regulation is largely exerted through a set of regulatory proteins, called transcription factors (TFs). These TFs regulate transcriptional activity via directly or indirectly interacting with DNA and their effect can be either positive or negative. An important feature of the TFs is that their activities are commonly (but not exclusively) modulated at the post-transcriptional level, such as phosphorylation or ligand binding, thus the TF activity (TFA) does not necessarily correlate with TF mRNA, or protein, levels. Furthermore, the TF-gene interaction is condition-dependent [1,2].

Transcriptome profiles are often modeled in a log linear fashion [3,4]:

(1) E=AP+Γ

where matrix **E**(*N *× *M *) represents the log ratio of gene expression of *N *genes in *M *microarray data, matrix **P**(*L *× *M *) describes the profiles of *L *hidden TFAs, while matrix **A**(*N *× *L*) defines the control strengths (CS) of TF to genes, and finally **Γ **is the unavoidable measurement noise. Matrix decomposition of the Eq. (1) does not provide a unique solution for **A **and **P **even with the same residual **Γ**, unless it is properly constrained.

Different methods have been developed to determine **A **and **P **uniquely under different assumptions. Principal component analysis (PCA) [5-7], assumes that the rows in the **P **matrix are orthonormal, while independent component analysis (ICA) [8-10] requires that the expression modes are statistically independent and non-Gaussian. Although these methods are useful in reducing the dimensionality, they do not provide TFAs because their assumptions do not reflect the underlying biological network. MatrixREDUCE [11,3,12] assumes that the control strength of the TF to its regulated gene is proportional to the number of occurrence of the TF motif in the promoter region of the gene, and the activities of the transcriptional regulators associated with each motif are determined by regression analysis. This above assumption needs to be refined, particularly when genes in the network are regulated by multiple TFs. Moreover, the TF-gene interaction is condition-dependent [1,2], which makes the problem even more challenging.

### Network Component Analysis

Network component analysis (NCA) [3,4,13] is a method that also aims to solve both **A **and **P **in Eq. (1) by incorporating TF-gene interaction information as constraints. Differing from MatrixREDUCE, NCA quantitatively determines both activities of the transcriptional regulators and the control strengths (CS) of TF-gene interactions. The network topology also needs to satisfy some criteria [3] to guarantee uniqueness of solution for the matrix decomposition of Eq. (1)--networks that meet these three criteria are termed NCA-compliant. The conditions necessary for NCA-compliance are [13,14]: (i) a selected TF must regulate *N *- *L+*1, or less, genes, where *N *and *L *are the number of selected genes and TFs in the network, respectively; (ii) the regulon of one TF cannot be a subset of another TF; and (iii) the number of TFs regulating a gene must be less than or equal to the number of transcriptome measurements (*M*). In the event that these necessary conditions are not met, the regulatory network can be broken into smaller NCA-compliant subnetworks for analysis. In higher eukaryotes, where a single gene can be regulated by a large number of TFs, criterion (iii) may not be met. To facilitate the analysis of regulatory networks that violate criterion (iii), we develop a data-augmentation algorithm below.

NCA correctly identifies the TFA profiles, if TF-gene relationships are provided. However, it is virtually impossible to obtain condition-dependent TF-gene connectivity information at the genome level for every condition of interest. Although there are extensive databases of regulatory interactions created from ChIP-Chip studies [2], DNA adenine methyltransferase identification (DamID) [15], or manual- and computer-aided literature mining [16,17], these databases may contain numerous false positives for a condition of interest. Using connectivity input with false positives may lead to inaccurate estimations of the control strengths (i.e. **A **matrix). And, in the case of a large percentage of false positive connections in a TF's regulon, the estimated TFA may be highly inaccurate. To remove the influence of false positive connectivity on data analysis, Yu and Li [18] used two sets of TF-gene interactions: a set of low-confidence interactions and a subset of higher-confidence interactions derived from biomedical literature and ChIP data respectively. The algorithm started by using the higher-confidence interactions as the constraints for the decomposition, and then added a new TF-gene edge per iteration from the lower-confidence set until the residual satisfied a specified criterion. Yu and Li tested the approach with *Saccharomyces cerevisiae *expression data measured under normal and stress conditions. Unfortunately, in the case of mammals, the number of high confidence TF-gene interactions is, currently, limited thus only a few TFs may be analyzed by the method of Yu and Li. To build accurate models of regulation in mammals, it is essential to develop a strategy that incorporates as many available TFs as possible into the analyzed network, while screening out the false TF-gene interactions. In this study we develop an iterative algorithm to trim the TF-gene interaction network to suit the specific condition of interest. The method integrates NCA with stepwise regression, which is a statistical method for model selection, so that the trimmed network will only contain relevant edges. We then apply a permutation test to identify key TFs and their target genes in the trimmed network. Finally, we employ a statistical sampling method to generate additional data from the existing data; this additional data is used when the original data set fails to meet the third criterion for NCA-compliance. The generation of additional data increases stability of NCA for complex regulatory networks, but does not lead to overfitting because the stepwise regression component of our network trimming approach eliminates any redundancy.

## Results

**Figure 1:**
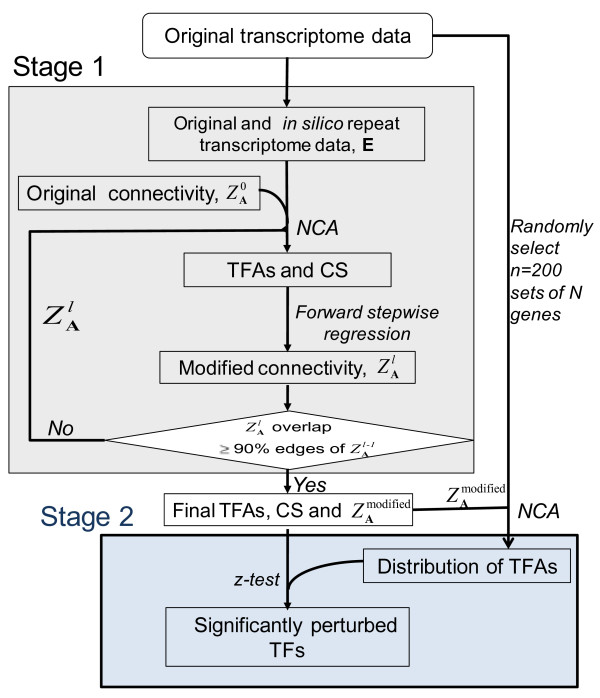
**A schematic overview of the method**. The TFA perturbations are estimated in an iterative fashion that trims unimportant edges from the regulatory (connectivity) network. If the data set is not NCA-compliant, additional data are generated *in silico *before estimating the TFA perturbations. Then, the significance of the TFA perturbations is assessed using the z-test and null distributions created by random sampling of expression data across the whole genome.

The method developed here includes two stages (Figure 1): (1) trimming the network by an iterative algorithm that integrates NCA with forward stepwise regression, and then (2) identifying the most important key TFs and target genes by permutation tests. To stabilize the NCA algorithm when insufficient data are available, a statistical method is employed.

### Trimming the network by the iterative NCA-stepwise regression algorithm

**Figure 2:**
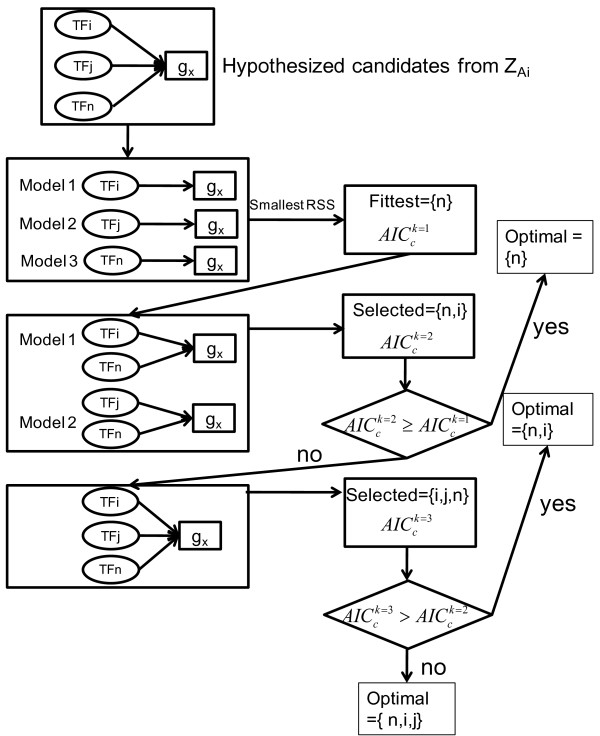
**Flow diagram illustrating the modified forward stepwise regression method as applied to a gene (g_x_) which has three candidate regulators (TF_i_, TF_j_, TF_n_)**.

Edge-trimming of the network is accomplished using the forward stepwise regression method (Figure 2). The expression of individual genes is modeled in Eq. (1) as:

(2) $$e_{i}=\sum_{j\in C_{i}}a_{ij}p_{j}+\Gamma$$

where, C_i _is the set of TFs regulating gene *i*. Only TFs with a non-zero connection to gene *i *are included in C_i_. Here, we are interested in estimating *a*_*ij *_for a given expression profile *e*_*i*_.

To estimate *a*_*ij*_, forward stepwise regression requires the explanatory variables **p**'s (i.e. TFAs). The TFAs are first estimated from NCA using the full connectivity pattern $Z_{A}^{0}$, which includes false positive connections. We have previously shown that most TFA profiles obtained by NCA are very robust [13,19] when the fraction of false positive edges in Z_**A **_was below a certain level [19]. By assuming that the false positive edges in the full connectivity pattern $Z_{A}^{0}$ exist at the moderate level which does not affect the TFAs, the trimming algorithm (Stage 1 in Figure 1) is summarized below:

i) Calculate TFA based on the full set of $Z_{A}^{0}$ using NCA. (Note that the superscript of Z_A _indicates the number of passages through the iteration.) The TFA for each TF is used as the explanatory variable in equation 2. Performing NCA with the Tikhonov regularization algorithm [4] is recommended because of its stability to ill-conditioned matrices generated during the bi-linear optimization phase.

ii) Initially, assume that only one TF regulates the gene, or *k *= 1, and generate different models using all the TFs in $C_{i}^{0}$ which is the list of indices of non-zero elements defined by row *i *of $Z_{A}^{0}$. The coefficient *a*_*ij *_and residual sum of squares (RSS) of all the models are calculated by linear regression using the TFA determined from step (i).

iii) Select the model having the smallest RSS.

iv) Calculate the modified Akaike information criterion [20], AIC_c_:

(3) $$AIC_{c}^{k}=M\log2\pi {\hat{\sigma }}^{2}+\frac{M\left(M+k\right)}{M-k-2}$$

where *M *is the number of columns in matrix **E**, and ${\hat{\sigma }}^{2}=\frac{RSS}{M}$ is the estimated variance of fitting errors computed from maximum likelihood. The $AIC_{c}^{k=1}$ is calculated by Eq. (3) for the chosen model. We use the modified AIC_c _instead of the F statistic because it includes a penalty term for small values of *M *to avoid overfitting. AIC_c _also does not require a user-input threshold as used in the F test.

v) The procedure from (ii) to (iv) is repeated for *k = k+1 *by incorporating an additional TF, from C_i_, to the best model identified in (iii). The $AIC_{c}^{k}$ is computed at the end of each iteration and compared with one in the previous iteration to decide if the new model better explains the *e*_*i *_than the model from the previous iteration. If $AIC_{c}^{k}\geq AIC_{c}^{k-1}$, the procedure is stopped, and only the *k*-1 TFs from the previous iteration are used to explain ***e***_***i***_.

vi) Steps (ii) to (v) are executed for each gene. When finished, the method identifies from $C_{i}^{0}$ a subset of TFs, defined as $C_{i}^{\text{triml}}$, to describe the expression data for gene i. In other words, only the strong interactions between gene *i *and the TFs in the final model are retained in the trimmed network. Figure 2 illustrates in detail how the method is applied to a gene that is initially thought to be regulated by three TFs. After this step the original network structure $Z_{A}^{0}$ is modified to $Z_{A}^{trim\;l=1}$.

vii) Compare updated $Z_{A}^{trim\;l=1}$ with the previous one $Z_{A}^{trim\;l-1}$. If the number of retained edges is greater than 99%, or any practical value suitable to the specific application, then the iteration stops and the final connectivity is defined as $Z_{A}^{final}$. Otherwise repeat step (i) to (vi) to obtain new $Z_{A}^{trim\;l+1}$.

The steps for trimming edges (from step (ii) to (vi) ) are encoded by a Matlab function in Additional file 1.

### Approach validation with synthetic data

**Figure 3:**
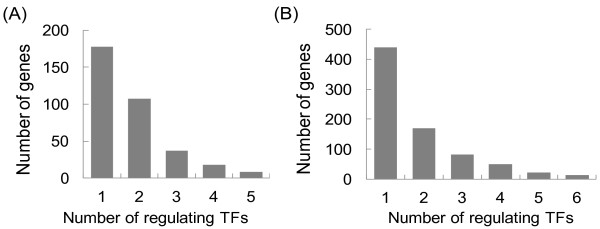
**The distributions of the number of TFs regulating a gene in the (A) synthetic and (B) mouse networks follow power law decay**.

To assess the performance of our algorithm we defined a synthetic regulatory network with "true" CSs and "true" TFAs, and then explored the effect of noise, false connectivity, and the number of independent data sets on the CS and TFA estimates from our algorithm. The method used to generate the synthetic network is described in Additional file 2. In brief, we designed a synthetic network of 348 genes regulated by 20 TFs that mimics key characteristics of mammalian regulatory networks, such as the murine transcription network (described below). A prominent feature of the complex mammalian regulatory networks is that genes are often regulated by multiple TFs. The complexity of a connectivity network is described by the distributions of the number of TFs regulating a gene; the distributions of both the synthetic (Figure 3A) and the mouse network (Figure 3B) follow a power law distribution.

We benchmarked our algorithm by comparing the deconvolution results to the true values as a function of a variety of key factors. We explored the robustness of our algorithm as a function of noise (**Γ**), independent data sets (r), and the false connection rate (FCR). The FCR is defined as the ratio of the false connected edges (nonzeros in initial Z^0^, but true CS = 0) over the number of true connected edges. We evaluated the algorithm at two different noise levels (0.1 and 0.5), four data set sizes, *r *= 3, 6, 9, 15 and 21, and four FCRs (0.18, 0.2, 0.25 and 0.30). Examining our algorithm as a function of r could guide in selecting an appropriate number of microarray experiments for a particular research problem. Due to high costs and low sample sizes that are often associated with studies involving mammals, it is important to know the required sample size. Note that in the *r *= 3 case, genes regulated by more than 3 TFs would violate NCA criterion (iii) and the regulatory networks for these genes would be underdetermined. Although this problem is prevalent when analyzing mammalian transcriptome data, it may be overcome by generating additional data *in silico *(See Methods and Additional file 2).

We first assessed the performance of the iterative NCA-stepwise regression algorithm for (1) the speed of convergence, and (2) the accuracy of reconstructed TFAs and expression under various noise levels and numbers of independent data in a set at the FCR = 0.20. The iterative algorithm converged at the second iteration, *l *= 2, with less than 1% of the edges trimmed in the second generation. After the first iteration (Z^1^), the number of edges in the trimmed connectivity network decreased around 23% to 30% compared to the original network (Figure S1A in Additional file 2), but the relative fitting errors (i.e. square root of the ratio between residual sum of squares and signal sum of squares) between raw and reconstructed expressions (Additional file 3) did not change much after the first iteration. This result implies that forward stepwise regression with modified AIC robustly removed most of the irrelevant edges from the network structure in the first iteration. Even though in some cases up to 30% of the edges were eliminated in the final networks, the final NCA-derived TFAs in all cases were nearly identical to the initial derived TFAs and to the "true" signals. The signal-to-noise ratio (SNR) was used to measure how good the derived TFAs were compared to the "true" values (Figure S1B in Additional file 2).

The main aim of the algorithm was to eliminate the false positive TF-gene interactions in the initial network. The false positive connections represent condition-specific interactions that are not relevant in the condition of interest as well as errors in the network. We evaluated the recovery of the edges at two levels: all and important TF-gene interactions. We focused on this qualitative aspect because a strong TF-gene interaction indicates that the gene's expression profile may serve as a biomarker for the activity of the respective TF in similar conditions. An important edge between a TF-gene pair will have a large |CS|, thus indicating that the gene's expression is highly sensitive to variations of the respective TFA. Knowing which genes in a TF's regulon are receiving a relatively strong signal will guide interpretation of the effects of pleiotropic global regulators such as p53 that have a wide range of influence. In this analysis, we arbitrarily classify a TF-gene interaction as important if its |CS| is in the top 30% of its regulon.

**Figure 4:**
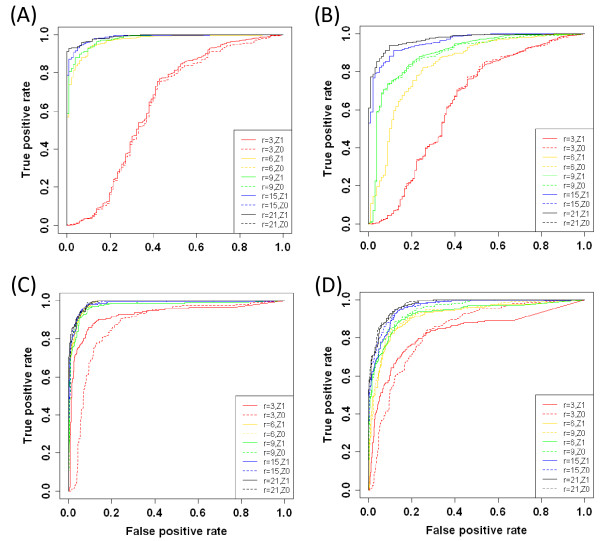
**ROC curves of the reconstructed edges at different r and noise levels**. ROC curves of original Z^0 ^(dashed lines) and trimmed Z^1 ^(solid lines) are used to assess the performance of network trimming on a synthetic data set at false connection rate = 0.20 for low (A&C) and high (B&D) noise levels, 0.1 and 0.5 respectively. Performance was assessed for various numbers of data points; r = 3 (red), 6 (gold), 9 (green), 15(blue), and 21(black). The rates on the axis are calculated based on counting all edges (A&B) or only important edges (C&D).

To visualize the increased performance imparted by our network trimming algorithm, we've generated Receiver Operating Characteristic (ROC) curves for the untrimmed and trimmed network as a function of noise (Figure 4). The ROC curves (Figure 4A &4B) indicate that algorithm performance increases with the number of experiments, and performance is optimal when the number of the data points is high enough (r ≥ 6) to ensure the NCA criterion (iii) satisfied. Not surprisingly, the performance was better at low noise level (4A vs. 4B) at which ROC curves of NCA compliant networks were discrete from that of incompliant network. When assessing performance based on recovery of all the true-positive interactions in the network, there did not appear to be a substantial benefit conferred by network trimming. However, when we focus on the important interactions, the trimming algorithm increases overall performance, especially for low experiment numbers (r = 3).

**Figure 5:**
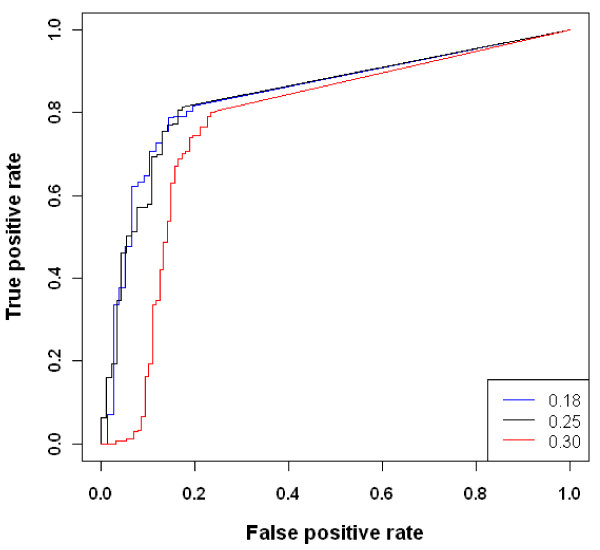
**ROC curves illustrating the performance of the algorithm at different FCR = 0.18 (blue), 0.25 (black) and 0.3 (red) at r = 3 and noise = 0.5**.

Finally, we explored the robustness of the algorithm at different FCRs. We focused on the worst case scenario, low number of experiments and high noise, and three FCRs = 0.18, 0.25, and 0.30. The performance based on all edges was quite robust for FCR < = 0.25, and with decayed performance evident at FCR = 0.30 (Figure 5). A similar trend was also observed when exploring only important edges.

### Exploring biological data

We used gene expression data from wild type (WT) mice [21] to test the applicability of our method. Gene expression was measured by Affymetrix chips in four different setups: 12 and 48 hours after treating mice with either placebo or RAD001 (everolimus)--a derivative of rapamycin. RAD001 binds to the immunophilin FK Binding Protein-12 (FKBP-12) to generate an immunosuppressive complex that binds to and inhibits the activation of the mammalian Target of Rapamycin (mTOR), a key regulatory kinase. Data were downloaded from NCBI's Gene Expression Omnibus (accession id GSE1413). Three different configurations were employed to compare the effect of RAD001 treatment vs. a placebo on gene expression at different time points (t = 12 and 48 hours) as well as between time points (i.e. 48 vs. 12 hrs) after RAD001 treatment. To explore the value of our data augmentation algorithm, the expression data were pre-processed in two different manners: with or without using our data augmentation algorithm (Section 4.2; Additional file 2). In brief, in the augmented expression data the three log ratios were generated based on averages of replicated samples and then augmented 9 times, while in the "raw" replicated expression data, the log ratios were established between RAD001 replicates and averages of placebo replicates at the respective time points and between the averages of RAD001 replicates at two time points to also obtain a total of 12 ratios for each gene.

The connectivity matrix $Z_{A}^{0}$ was constructed from the transcriptional regulatory element database TRED [16] of Cold Spring Harbor Laboratory. In this study a TF-gene interaction was assumed to exist if the information for binding quality in TRED was defined as "known", "likely", or "maybe". The analyzed network contained 778 genes regulated by 70 TFs. The trimmed network structure and its regulatory signal were first derived by the iterative algorithm. The permutation approach described in Methods (Section 4.1) was then used to identify significantly perturbed TFs under RAD001 treatment. In this analysis the TFA null distributions were built from *n *= 270 randomly scrambled data sets (E_random_) using the trimmed connectivity matrix obtained in the iterative algorithm. The TFs were identified as being significantly perturbed if the p-values of a two-tailed z-test were less than 0.1 based on the simulated data (Figure S2 in Additional file 2) in which a large percentage (~22%) of TFAs were set to be perturbed. The ROC curves (Figure S2) indicate the optimal cut off p-values for the trimmed and original networks around 0.1-0.26.

We first assessed the advantage of data augmentation by examining the relative fitting errors between the reconstructed and input expression data. Although both augmented and raw replicated expression data were the same size and were analyzed by the same connectivity matrix, the relative fitting error when using the augmented data set was 0.4 whereas the raw data had a relative fitting error of 0.61. Another potential disadvantage of using the raw data was that more edges were removed from the network during the trimming process (35% for raw vs. 19% for augmented). Note, that in the simulation at the high noise level (Figure 4D) the significant removal of edges would decrease the TPR. When the raw data were used expected interactions, such as Hif1a-*Eno1*, were trimmed from the network. However, the removal of edges did not drastically affect the TFA profiles. The average of correlation coefficients for each TFA between the first and second iteration were 0.98 and 0.96 for the raw replicated and augmented data, and that of final TFAs between two scenarios was 0.87. In the following section, we focus on results obtained with the augmented expression data due to the higher TPR.

Similar to the synthetic examples, trimming of the mouse network converged after two iterations with about 75% of the original edges retained. Trimming the network allowed us to identify key interactions that were obscured when employing the untrimmed network. For example, when the data are analyzed using the trimmed network *Eno1 *is predicted as an important target of Hif1a with a |CS| = 1.6 (ranked at the 7^th ^among 37 Hif1a regulated genes). *Eno1 *is known to be regulated by Hif1a [22], and has been used as a reporter for Hif1a activity [23,24]. However, when analysis of this network is performed without trimming, this interaction is obscured (|CS| = 0.9 and ranked as 17^th ^in the regulon).

Additionally, trimming the network allowed us to correctly infer the direction of regulation of TFs to genes. For example, expression of the multidrug resistance phosphoglycoprotein (*Abcb1b*) is known to be activated by Trp53 [25]. Besides Trp53, several other TFs such as Cebpb, Hif1a, Tcfap2a, and Pgr are also indicted as *Abcb1b *regulators by TRED. Without network trimming, the NCA implied that *Abcb1b *was repressed by Trp53. However, after the weak interactions between other TFs and *Abcb1b *were removed, *Abcb1b's *expression was correctly predicted to be activated by Trp53 and repressed by Tcfap2a (Additional file 4). The incorrect prediction with the initial network was due to overfitting problems arising when many regulators are used to explain the variation of few data points.

**Figure 6:**
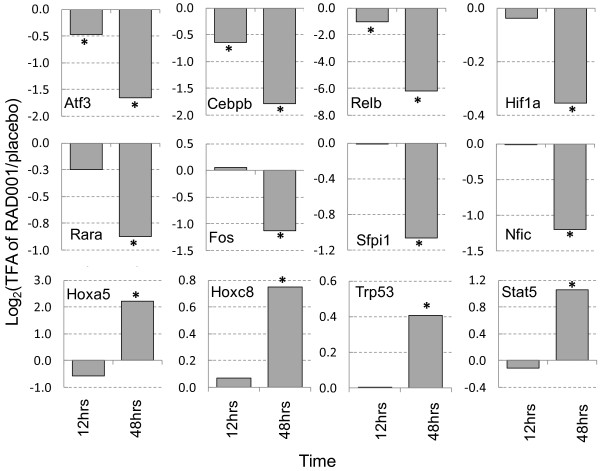
The TFA profiles of the 12 significantly (^*^p < 0.05) perturbed TFs in WT mouse prostate at 12 and 48 hours after rapamycin injection.

In addition to identifying important TF-gene interactions in the RAD001 response, we were interested in exploring how the network response is integrated; thus, we explored relationships between perturbed TFs. The TFAs of 17 TFs (Atf3, Cebpb, RelB, Hif1a, Rara, Fos, Sfpi1, Nfic, Hoxa5, Hoxc8, Trp53, Stat5b, Atf1, Nfkb1, Myb, Smad7, and Wt1) were significantly (p < 0.1 two-tailed z-test) perturbed in prostate tissue of wild type mice at 12 or 48 hours after treating mice with the mTOR inhibitor RAD001. TFA profiles for the 12 TFs (Atf3, Cebpb, Relb, Hif1a, Rara, Fos, Sfpi1, Nfic, Hoxa5, Hoxc8, Trp53, and Stat5b) with p-values < 0.05 are shown in Figure 6. The p-values were then adjusted by the Benjamini and Hochberg method to account for multiple hypotheses testing. The TFAs of eight TFs (i.e. Atf3, Cebpb, Hoxa5, Hoxc8, Nfic, RelB, Sfpi1, and Stat5b) were consistently identified as significantly (p-value < 0.1) perturbed after 48 hours. Among the 17 significantly perturbed TFs, Atf3, Cebpb, and RelB responded earlier at 12 hours after treatment, and were down-regulated further after 48 hours, while the remaining TFs were only significantly perturbed at 48 hours. RAD001 treatment decreased all of the TFAs except Hoxa5, Hoxc8, Trp53, and Stat5b. The perturbation of Hif1a was confirmed in the original paper [21], while Atf1 [26], Cebpb, and Nfkb1 [27] were identified as targets of rapamycin in different human cell lines. By mapping the perturbed TFs to KEGG database, we found the set of perturbed TFs was enriched for three pathways: acute and chronic myeloid leukemia, and MAPK signaling pathways with the p-values of Fisher's exact test less than 0.1 and fold enrichment being greater than 6. Recently it has been found that RAD001 activates the MAPK pathways through a PI3K-dependent feedback loop in human cancer [28].

**Figure 7:**
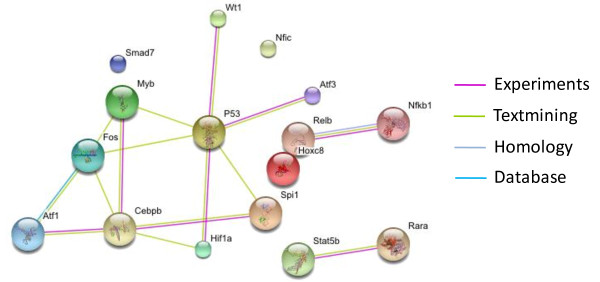
**The majority of the RAD001-sensitive TFs identified by NCA with network trimming interact with each other as determined with the STRING toolbox**.

To identify relationships between the 17 significantly perturbed TFs we subjected them to a variety of informatics analyses. These TFs were first subjected to signaling pathway analysis to see if they shared any common upstream pathways. Three signaling pathways commonly used by these TFs were identified with the Functional Annotation Tool box from DAVID [29]: (1) the JNK/p38 MAPK signaling pathway, which involves Trp53, Nfkb1, and Fos as its down-stream targets, (2) the acute and (3) chronic myeloid leukemia pathways which contain Sfpi1, Stat5b and Rara. To determine if any of the TFs interact each other, we used the STRING [30] toolbox to identify gene/protein interactions (Figure 7). STRING identifies interactions based on evidence from experimental data, homology, text mining, etc. Experimental data indicate that Cebpb interacts with Myb, Sfpi1, and Atf1 [31-33], that RelB interacts with Nfkb1 [34], and Trp53 (or p53) also associated with Hif1a, Atf3 and Wt1 [35-37]. In brief, these key TFs are highly connected to each other by either sharing common signaling pathways or through protein-protein interactions.

## Discussion

Although NCA can deduce a TFA, it may not accurately estimate the strength of the TF-gene connectivity because the initial network connectivity map may contain a number of false positive TF-gene connections. These false positive connections, typically, arise from either a false prediction or a condition-dependent TF-gene interaction. The goal of network trimming is to identify the *key *TFs and their regulated genes from the initial network connectivity map containing a moderate level of false connections. Our algorithm trims superfluous connections, so that the important connections will standout. Removing connections that are not relevant to the condition of interest is of crucial importance when dealing with pleiotropic global regulators, such as p53.

We, also, present a data augmentation algorithm that expands the applicability of NCA to the study of mammalian transcription networks which frequently cannot be analyzed by NCA due to a dearth of data. Besides stabilizing the NCA numerical decomposition, generating additional data *in silico *allows more genes to be analyzed thus reducing the possibility that important genes will not be analyzed. And, we show that our augmentation algorithm circumvents the problem of a high degree of biological noise in the real biological data, in which the estimated CSs become artificial products of fitting noise rather than reflecting the strength of association between TF-gene, and that noise leads to a lower TPR for important TF-gene interactions as was demonstrated with the synthetic and biological examples. One potential drawback of using our data augmentation algorithm is that some biological variation in TFAs may be averaged out. To assess if any biological variation was averaged-away during data augmentation, it is possible to analyze the raw data with the trimmed network. Our method rules out the influence of overfitting that can arise from data augmentation; the stepwise regression component of our network trimming approach eliminates redundancy such that the number of regulators regulating each individual gene in the final network is constrained by the number of available data points.

The performance of our approach does not depend on the size of the network, but rather features of the network structure such as: how many TFs regulate a gene, number of genes regulated by a TF (i.e. regulon size), overlap between regulons, etc. Because our goal is to extend NCA to the analysis of complex mammalian networks, the simulated network structure was constructed similar to the murine network, and incorporated the aforementioned features at different levels. For example, the simulated network regulon size varies from 5 to more than 80 genes. Various regulation patterns are also covered in this network: (i) Some TFs mostly regulate their genes alone (e.g. only 20% of regulated genes are shared with other TFs), while some TFs often strongly overlap with other TFs in regulating genes (e.g. up to 80% of regulated genes are shared with other TFs).

Our analysis indicates that the algorithm is beneficial in the case that the number of experiments, r, is so small that prior incarnations of NCA cannot be used. When few data sets are available at the time of analysis, our method can be used for screening the important TFs which will aid in the design of further experiments for studying their regulated genes. The transcriptome data from the new experiments can be added to the existing datasets for another round of analysis to improve the prediction further.

We employed our approach to analyze transcriptome data taken from wild type mice treated with RAD001 [21] (Figure 6). RAD001 is an inhibitor of mTOR, which is a serine/threonine protein kinase that regulates cell growth, proliferation, and motility through both transcriptional and translational regulation. Among the 17 key TFs identified as targets of rapamycin by the method, Hif1a, Cebpb, Nfkb1, and Atf1 are confirmed by literature as rapamycin-related TFs. FoxO is a TF that has recently been reported [38] to be involved in mTOR signaling, but unfortunately it was not included in the analyzed network because it was not in the database from which the original connectivity matrix was derived. Our algorithm is only able to identify target TFs from a given network, and it is beyond the scope of the approach for identifying TFs without information regarding their regulated genes.

Since 13 out of 17 TFs have not been identified in the literature as being related to RAD001, we searched for potential links in known signaling pathways and reported, or predicted, protein interactions. If they are perturbed by RAD001 treatment, it is plausible that they share common signaling pathways or interact with each other. Pathway analysis and protein interaction analysis based on literature mining indicated the presence of evidence supporting interactions between a number of the TFs (Figure 7). Taken together, the NCA results and pathway/interaction analyses highlight additional components of the RAD001 response that may be of interest when developing targeted therapeutics.

Like the Gibbs sampling method [1], our method aims to identify the most reliable edges in the NCA compliant network. However, the novelty of our strategy is that it trims the network so that we can analyze a network with more genes and less data points than the Gibbs sampling method. Thus, our method can start with a NCA incompliant network whereas the Gibbs sampling method requires the initial network as NCA compliant, so the Gibbs sampling method is not suitable for interpreting a large number of mammalian data sets. Overall, we've provided an approach that facilitates the application of NCA to complex mammalian networks with limited data.

## Conclusions

The advantage of our new algorithm, relative to the original NCA, is that our algorithm can identify the important TF-gene interactions. Identifying the important TF-gene interactions is crucial for understanding the roles of pleiotropic global regulators, such as p53. Also, our algorithm has been developed to overcome NCA's inability to analyze large networks where multiple TFs regulate a single gene. Thus, our algorithm extends the applicability of NCA to the realm of mammalian regulatory network analysis.

## Methods

### Statistical permutation tests for identifying perturbed TFs

The TFA profiles derived from NCA describe the variation in TFAs for different experimental conditions compared to the respective reference states. Because the TFAs and CSs of each TF in NCA is normalized by its own scaling factor defined in [3], we cannot determine which TFs are significantly perturbed based solely on TFA values. Therefore, we need to develop a method to assess the statistical significance of the derived TFAs and CSs. To assess the probability that the NCA-deduced network, TFAreal, could have arisen by chance we employ the following procedure:

(i) The expression data matrix E is row randomly selected from expression measurements of the whole genome whose size is often larger than that of the analyzed network to yield E_random_, but the connectivity remains the same. This procedure in effect randomly assigns gene expression data to each gene from the population of expression data while keeping the transcriptional network unchanged.

(ii) NCA is carried out using $Z_{A}^{final}$ obtained by the above network trimming algorithm (Section 2.1) and E_random _from step (i). The signs (i.e. positive or negative) of TFAs in the random network, TFA_random_, are assigned based on TFA_real _because they are not considered in NCA normalization [3]. For example, if the Pearson correlation coefficient between TFA profiles of the random and real network is less than 0, the TFAs and CS of that TF are re-scaled by -1. This allows the TFA profiles in TFA_random _to become as similar to those of TFA_real_, and removes any bias caused by sampling the genes from the whole genome.

(iii) Steps (i) and (ii) are repeated for *n *≥ 200 random networks to generate null distributions of TFAs.

(iv) The p-values of TFAs derived from the compatible network are calculated from z-tests using the median and median absolute deviation (MAD) of the constructed null distributions.

### Stabilization of NCA algorithm

When the number of independent data is low, NCA criterion (iii) may be violated [13], causing numerical instability. To remedy this problem, we generate *in silico *data that behave similar to the biological data. The *in silico *data do not impact the final NCA solutions, since the effect of data generation is eliminated by linear stepwise regression. The *in silico *data are generated by assuming that the original log expression ratio *E*_*ij *_of gene *i *in the biological condition *j *has a normal distribution of *N *(*μ*_*ij*_, $\sigma_{ij}^{2}$). Since the mean *μ*_*ij *_and variance $\sigma_{ij}^{2}$ are not available for the existing dataset composed of a few data points, we then have to make further assumption that: (1) *μ*_*ij *_= *E*_*ij *_, which is the original gene expression data and (2) $\sigma_{ij}^{2}\approx \mu_{ij}^{2}\sigma^{2}$ where *σ*^2 ^is the variance of E after subtracting expression of each gene by its own average. The procedure is described by the following:

(i) E_norm _is generated by subtracting the original log expression ratios of each gene by its mean *μ*_*i *_.

(ii) The variance *σ*^2 ^of E_norm _is calculated

(iii) Each repeat data point ${\tilde{E}}_{ij}$ is randomly sampled from the normal distribution *N *(*μ*_*ij *_= *E*_*ij*_, $\sigma_{ij}^{2}\approx \mu_{ij}^{2}\sigma^{2}$). This step is repeated two three times to obtain 2-3 new repeat datasets.

NCA and network trimming are then applied to the expression data including both the existing and additional data. A rule of thumb for realizing NCA criterion iii, is that the total number of data should be approximately twice the maximal number of TFs regulating a gene.

Finally, the signal-to-noise ratio (SNR) is used to evaluate the ability of our algorithm to deduce the correct network structure. In this case it is equivalent to the ratio between the sum of squares of the true signals over that of errors between the true and reconstructed signal, and is usually expressed in terms of the logarithmic decibel scale as:

(4) $$SNR\left(dB\right)=10\log_{10}\left(\frac{\sum_{j,k}TFA_{jk,true}^{2}}{{\sum_{j,k}\left(TFA_{jk,true}-TFA_{jk,derived}\right)}^{2}}\right)$$

where TFA_jk _is the signal of TF *j *at condition *k*.

## Authors' contributions

LMT and JCL designed the study. LMT performed the analysis. LMT, JCL, and DRH interpreted the results and wrote the manuscript. All authors read and approved the final manuscript.

## Supplementary Material

Additional file 1 **The M file encodes Matlab function for trimming edges by stepwise regression**. Click here for file

Additional file 2 **The document file provides additional information of the methods and results**. Click here for file

Additional file 3 **The excel file provides the network structure and fitting errors of the synthetic network**. Click here for file

Additional file 4 **This excel file provides all data of the real biology network**. Click here for file
