# Supplementary material for: Trimming of mammalian transcriptional networks using network component analysis
Source: BMC Bioinformatics. 2010 Oct 13;11:511. doi: 10.1186/1471-2105-11-511 (PMC2967563; doi:10.1186/1471-2105-11-511)
Supplement: Additional file 2 — The document file provides additional information of the methods and results. [file 1471-2105-11-511-S2.DOC]

# Supplementary Information

## Synthetic network generation

A synthetic network structure was designed to mimic key characteristics of mammalian regulatory networks. A prominent feature of mammalian regulatory networks is that genes are often regulated by multiple transcription factors (TFs). The synthetic network had 348 genes regulated by 20 TFs. The number of TFs regulating a gene followed the power law and went up to 5. The true CS values were first randomly generated based on the original connectivity $Z_{\mathbf{A}}^{0}$, provided in the Additional file 2, and some of them were decreased to or set to zero to generate insignificant and false edges in the original connectivity to get the final matrix **A** of true CS (see Additional file 2). The regulatory signals of 20 TFs (i.e. matrix **P**) were next randomly generated. Some of them were set to be significantly higher than the rest for later testing our statistical approach of determining the significant TFAs. Expression ratios (**E**) were then calculated by Eq. in the main text. In this study the noise,$\mathbf{\Gamma}$, had white Gaussian distribution with the noise level defined as the ratio between the powers of noise ($\mathbf{\Gamma}$) and true constructed expressions (i.e.$\mathbf{A}\cdot\mathbf{P}$).

We assessed the robustness of our algorithm as a function of noise ($\mathbf{\Gamma}$) and independent data sets (*r*). Two different noise levels, 0.1 and 0.5, were used to test the robustness of the approach to noise. We used four different data set sizes, *r* = 3, 9, 15 and 21. Note that in the *r* = 3 case, which was analogous to the real biological network and required generating additional data *in silico* (See Methods), we generated 6 additional data sets *in silico* thus providing us with a total of 9 data sets, which are sufficient to meet criterion (iii) for our synthetic network where the maximum connectivity was 5 TFs regulating 1 gene. To compare the TFAs from the augmented data with the true TFAs, we first reduced the column number of TFA_derived_ from 9 to 3 by averaging the dependent data.

For evaluating the false discovery rate in determining significantly perturbed TFAs, we generated the simulated whole genome network which provides the pool expression for sampling the genes for constructing the null distribution of TFA of the sub-network of interest. In this simulation, we supposed that the investigated subnetwork included 1/11 of total genes in the genome and regulated by 20 out of total 80TFs. Among 20 investigated TFs in 3 conditions, 22% (=13/(3*20)) of them were set to be significant perturbed. Therefore, besides the original network we generate addition expression of genes regulated by 60 TFs in r = 3 conditions. Some of TFAs were random selected and set as being significantly perturbed. The noise = 0.5 was added to all expression data. The null distribution of the simulated TFAs were constructed as described in Section 4.1 of the main text with n =200 random networks whose expression were selected from the pool of 348 investigated +3480 existed but non-interested genes. Figure S2 indicated that Z^1^ provided better sensitivity and specificity than Z^0^ at the same cutoff level of p-value. The optimal cut off value for the analysis, however, was quite high compare to the traditional cutoff values (e.g. 0.05).

**Pre-processing the real expression data**

We used gene expression data from wild type (WT) mice to test the applicability of our method. Gene expression was measured by Affymetrix chips in four different setups: 12 and 48 hours after treating mice with either placebo or RAD001 (everolimus)—a derivative of rapamycin. RAD001 binds to the immunophilin FK Binding Protein-12 (FKBP-12) to generate an immunosuppressive complex that binds to and inhibits the activation of the mammalian Target of Rapamycin (mTOR), a key regulatory kinase. Data were downloaded from NCBI's Gene Expression Omnibus ([http://www.ncbi.nlm.nih.gov/geo/; accession id GSE1413)](http://www.ncbi.nlm.nih.gov/geo/; accession id GSE1413) with the accession numbers GSE1413)  including four 12h placebo, six12h RAD001, four 48h placebo and five 48h RAD001 replicates. Three different comparisons were established for comparing expression between RAD001 and placebo treatment at different time points (i.e. t = 12 and 48 hours) as well as between time points (i.e. 48 vs 12 hrs) after RAD001 treatment. The expression data were pre-processed with and without the data augmentation algorithm to illustrate the benefit of the data augmentation algorithm. For the data augmentation algorithm, the downloaded data was preprocessed as follows: The data points for each probe were averaged over replicates and then converted to log ratios. Three log ratio datasets were generated for comparing expression between RAD001 and placebo treatment at different time points (i.e. t = 12 and 48 hours) as well as between time points (i.e. 48 vs 12 hrs) after RAD001 treatment. Gene symbols were used for gene identity; therefore, if a gene had multiple probe sets, the average of multiple probe sets was used as its expression. The data augmentation algorithm was then used to create nine *in silico* log ratio datasets (3 per log ratio) to raise the total number of datasets to 12. When the data augmentation algorithm was not used, the log ratios were generated between replicates of RAD001 treatment and mean of samples of placebo treatment at the corresponding time points to obtained six 12h and five 48h ratios. The last log ratio was generated between the average of 12h and 48h RAD001 treatment. Therefore, both augmented and “raw” replicated data had the same 12 data points.

**Reference**

**1. Majumder PK, Febbo PG, Bikoff R, Berger R, Xue Q, McMahon LM, Manola J, Brugarolas J, McDonnell TJ, Golub TR, Loda M, Lane HA, Sellers WR: mTOR inhibition reverses Akt-dependent prostate intraepithelial neoplasia through regulation of apoptotic and HIF-1-dependent pathways. *Nat Med* 2004, 10(6):594-601.**

Figure S1: The performance of network trimming on a synthetic data set*.*

(a) More edges are trimmed when there is more noise or less data available. (b) Despite decaying with increasing noise, or decreasing data availability, the signal-to-noise ratio (SNR) was strong for the cases examined.

Figure S2: ROC curves used to find the optimal cutoff p-value in determining the significant TFAs.

The p-values are calculated based on original Z^0^ (blue) and trimmed Z^1^ (red). The numbers along the curves are the respected cutoff p-values used.
